# Molecular and Cellular Studies Reveal Folding Defects of Human Ornithine Aminotransferase Variants Associated With Gyrate Atrophy of the Choroid and Retina

**DOI:** 10.3389/fmolb.2021.695205

**Published:** 2021-07-30

**Authors:** Riccardo Montioli, Giada Sgaravizzi, Maria Andrea Desbats, Silvia Grottelli, Carla Borri Voltattorni, Leonardo Salviati, Barbara Cellini

**Affiliations:** ^1^ Section of Biological Chemistry, Department of Neurosciences, Biomedicine and Movement Sciences, University of Verona, Verona, Italy; ^2^ Department of Medicine and Surgery, University of Perugia, Perugia, Italy; ^3^ Clinical Genetics Unit, Department of Woman and Child Health, University of Padova, Padova, Italy

**Keywords:** gyrate atrophy, pyridoxal phosphate, ornithine aminotransferase, pathogenic variant, vitamin B6

## Abstract

The deficit of human ornithine aminotransferase (hOAT) is responsible for gyrate atrophy (GA), a rare recessive inherited disorder. Although more than 60 disease-associated mutations have been identified to date, the molecular mechanisms explaining how each mutation leads to the deficit of OAT are mostly unknown. To fill this gap, we considered six representative missense mutations present in homozygous patients concerning residues spread over the hOAT structure. *E. coli* expression, spectroscopic, kinetic and bioinformatic analyses, reveal that the R154L and G237D mutations induce a catalytic more than a folding defect, the Q90E and R271K mutations mainly impact folding efficiency, while the E318K and C394Y mutations give rise to both folding and catalytic defects. In a human cellular model of disease folding-defective variants, although at a different extent, display reduced protein levels and/or specific activity, due to increased aggregation and/or degradation propensity. The supplementation with Vitamin B6, to mimic a treatment strategy available for GA patients, does not significantly improve the expression/activity of folding-defective variants, in contrast with the clinical responsiveness of patients bearing the E318K mutation. Thus, we speculate that the action of vitamin B6 could be also independent of hOAT. Overall, these data represent a further effort toward a comprehensive analysis of GA pathogenesis at molecular and cellular level, with important relapses for the improvement of genotype/phenotype correlations and the development of novel treatments.

## Introduction

Human ornithine aminotransferase (hOAT) is a pyridoxal 5′-phosphate (PLP)-dependent enzyme localized in the mitochondrial matrix, that catalyzes the δ-transamination of L-ornithine (L-Orn) and α-ketoglutarate (αKG) to glutamic-γ-semialdehyde and L-glutamate. The spontaneous cyclization of glutamic-γ-semialdehyde then generates the proline precursor pyrroline-5-carboxylate (P5C). The enzyme has a prominent role in L-Orn degradation and in proline synthesis, although some evidences also indicate its involvement in cell cycle regulation (Liu et al., 2019). The 439-residues chain of the hOAT precursor is encoded by the OAT gene located on chromosome 10p26, and then processed after cleavage of the mitochondrial targeting sequence to the mature protein (Inana et al., 1986). hOAT belongs to the Fold Type I family of PLP-enzymes (Shen et al., 1998) and in solution assembles as a tight homotetramer formed by two dimers that constitute the minimal functional unit of the enzyme (Montioli et al., 2017). Each subunit includes an N-terminal segment wrapping over the neighboring subunit, a large domain (residues 95–344) comprising the active site region and most of the subunit interface, and a C-terminal small domain (Shen et al., 1998). The active site lies at the junction between the two subunits of the dimer. PLP is bound through a Schiff base linkage with Lys292 and stabilized through hydrogen bonds, salt bridges and hydrophobic interactions with residues belonging to both subunits. The cofactor also exerts a structural role for hOAT, by promoting tetramerization and reducing the tendency to unfolding and aggregation typical of the apo-form (Montioli et al., 2017).

Inherited mutations on the OAT gene lead to gyrate atrophy of the choroid and retina (GA), a rare recessive disease characterized by the degeneration of the choroid and the retinal epithelium. Patients develop myopia during childhood, and show a progressive decrease in visual acuity from the 2° to the 3° decade leading to blindness within the 5° decade (Simell and Takki, 1973). In most GA patients cognition is unaffected (Wang et al., 2000). The deficit of hOAT leads to a 10-to-15 fold increase in the plasmatic L-Orn concentration, along with a small reduction in the levels of glutamate, glutamine, lysine and creatine (Valle et al., 1980; Hasanoglu et al., 1996). It is currently assumed that retinal damage is due to hyperornithinemia and to the increase in ornithine degradation products (Kaneko et al., 2007; Ohnaka et al., 2011), although the molecular mechanisms of GA pathophysiology are still unclear. No effective therapies are currently available, except for dietary restriction, which aims at reducing ornithine burden, and Vitamin B6 administration, which aims at increasing the plasmatic PLP concentration (Kennaway et al., 1980; Ramesh et al., 1988; Kennaway et al., 1989; Heller et al., 2017; Montioli et al., 2021).

**FIGURE 1:**
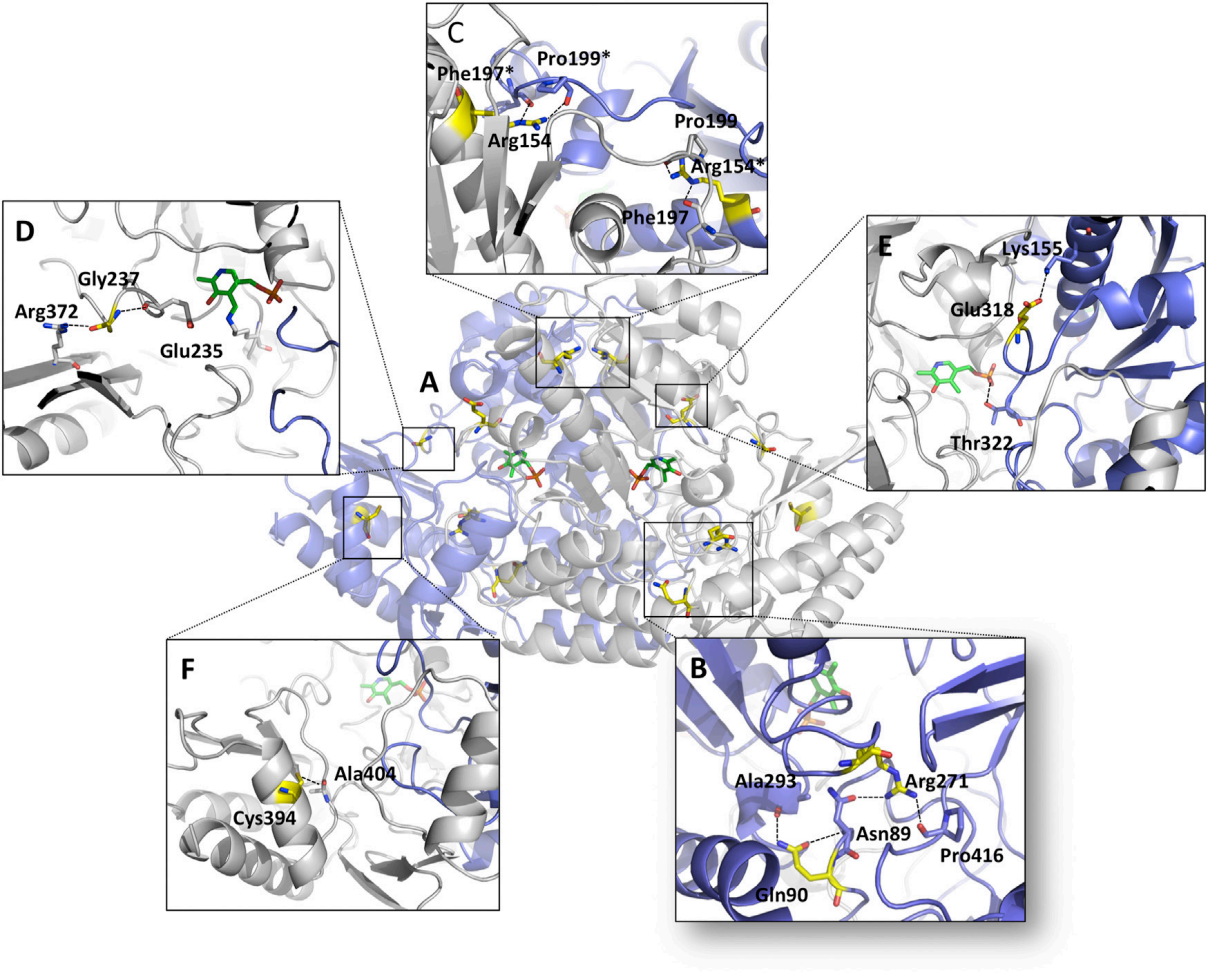
Overview of the mutation sites on the hOAT dimer. Ribbons representation of the OAT dimer; monomers are colored white and blue respectively, the residues subjected to mutation are highlighted in yellow and PLP molecules are represented as green sticks. **(A)** Global distribution of the mutation sites on OAT. Microenvironment and contacts of **(B)** Gln90/Arg271, **(C)** Arg154, **(D)** Gly237, **(E)** Glu318 and **(F)** Cys394 residues. *denotes residues belonging to the neighboring subunits. Image was rendered by Pymol software (Schrödinger).

The genetic cause of GA is known since 1973 (Simell and Takki, 1973), and 68 different disease-causing mutations in the OAT gene are currently deposited in the HGMD database, being missense changes the most common ones. To date, only two pathogenic variants have been characterized at protein level and/or in human cellular models of disease, revealing opposite molecular defects: the V332M, endowed with an unaltered intrinsic catalytic activity but affected by a folding defect that strongly reduces the intracellular levels of functional enzyme (Montioli et al., 2018), and the R180T, showing an altered structure but an almost complete loss of transaminase activity (Montioli et al., 2019). Considering that most disease-causing missense mutations involve residues that are spread over the enzyme structure, further investigations on the defect(s) caused by pathogenic mutations in hOAT would represent a useful tool toward a better understanding of GA pathogenesis. To this aim, we retrieved pathogenic mutations present in homozygous patients or in compound heterozygous with null mutations (Supplementary Table S1) and we specifically focused on mutations affecting 1) Gln90 of the N-terminal region, 2) Arg154, Arg271, Glu318 and Gly237 of the large domain, 3) Cys394 of the C-terminal small domain (Figure 1A).

We combined biochemical and bioinformatic analyses on the purified recombinant Q90E, R154L, G237D, R271K, E318K, and C394Y variants with expression studies in prokaryotic and eukaryotic cells. From our investigations, we deduced that 1) the R154L and G237D variants exhibit a dramatic loss of catalytic activity accompanied by a modest folding defect, 2) the Q90E and R271K variants display a remarkable folding defect responsible for increased degradation propensity, and 3) the E318K and C394Y variants show both catalytic and folding defects. In addition, we noticed that treatment of the folding-defective variants with Vitamin B6 does not significantly improve their expression levels and specific activity, possibly suggesting that effects unrelated to hOAT could be responsible for the clinical responsiveness of the patients.

## Materials and Methods

### Materials

L-Orn, α-KG, 2-aminobenzaldehyde, dimethyl sulphoxide, isopropyl-β-D-thiogalactoside, pyridoxine (PN), pyridoxamine (PM), pyridoxal (PL) were purchased from Sigma-Aldrich. 1,8-anilino-naphthalene sulfonic acid (ANS) was purchased from Molecular Probes. Growth media and additives were purchased from Gibco. PEGylated bis (sulfosuccinimidyl) suberate [BS (PEG) 5] was purchased from ThermoFisher. All other chemicals were of the highest purity available.

### Vector Construction

The vectors encoding the analyzed hOAT variants were obtained from the pOAT and the pLENTI6CMV-DEST vectors previously used for the prokaryotic and mammalian expression of hOAT, respectively (Montioli et al., 2017; Montioli et al., 2018). Each mutation was introduced by the QuikChange II site-directed mutagenesis kit (Agilent Technologies) using Pfu-Ultra polymerase and the oligonucleotides listed in Supplementary Table S2. All the mutations were confirmed by DNA sequence analysis of the entire ORF.

### In Silico Studies

Structural position and possible contacts of each mutated residue were evaluated by the PyMol software starting from the available crystal structure of hOAT (pdb file 1OAT). The in silico mutagenesis tool of PyMol was used to obtain the initial mutant structures. An energy minimization process in explicit solvent was carried out on the hOAT structure before and after the mutagenesis using the GROMACS v4.6.3 software. The Q90E, R271K, E318K and C394Y model structures underwent two minimization steps (with and without restraints), alternating Steepest Descent and Conjugate Gradient algorithms.

### Expression and Purification of Mutants

hOAT variants were expressed in *E. coli* and the cell lysate was treated as previously described (Montioli et al., 2017). The soluble fraction was loaded on a DEAE Sepharose 26/20 equilibrated with 20 mM sodium phosphate buffer, pH 7.6. A linear gradient from 20 to 180 mM sodium phosphate buffer, pH 7.6, was then applied. Under these conditions, OAT wild-type and all the pathogenic variants elute from the column between 110 and 160 mM sodium phosphate. Active fractions were then concentrated using an Amicon Ultra 15 unit (Millipore) and applied to a Superdex 200 XK 16/60 column (GE Healthcare) equilibrated in 50 mM HEPES, pH 7.4, NaCl 200 mM. Purified protein was concentrated and stored at −20°C. The purity of each preparation assessed by SDS-PAGE was >95%. The final yield of OAT WT, Q90E, R154L, G237D, R271K, E318K and C394Y variants were 35, 4, 26, 24, 7, 11 and 8 mg/L of liquid culture, respectively.

### Enzyme Activity Assays

hOAT activity was determined by a spectrophotometric assay based on the measurement of the dihydroquinazolium derivative of P5C after incubation with 2-aminobenzaldehyde as previously described (Montioli et al., 2017). The kinetic parameters for the overall transamination of the pair L-Orn/αKG were determined by incubating the purified protein in the presence of 100 µM PLP and by varying the substrate concentration at a fixed saturating cosubstrate concentration. Data were fitted to the Michaelis-Menten equation. In the case of the C394Y variant, data were fitted to the following equation:

$$\frac{v}{Et}=\frac{kcat\;}{\left(1+\left(\frac{\text{K}M}{\text{S}}\right)+\left(\frac{\text{S}}{\text{K}i}\right)\right)}$$ (1)

where E_t_ is the total enzyme concentration, *k*_*cat*_ the maximum velocity, S the substrate concentration, K_M_ the apparent Michaelis-Menten constant, and K_i_ the dissociation constant for the inhibitory ternary complex. To measure the intracellular hOAT transaminase activity, 100 μg of the soluble cellular lysate were incubated in 50 mM HEPES, pH 8.0, 150 mM NaCl and 100 μM PLP at 25°C with saturating concentration of L-Orn (100 mM) and α-KG (50 mM) for 15–60 min.

### Spectroscopic Measurements

Absorbance, Near-UV CD and fluorescence measurements were carried out in 50 mM HEPES containing 150 mM NaCl, pH 8.0, Far-UV CD spectra were registered in 10 mM HEPES pH 8.0, plus 15 mM NaCl. All measurements were performed at 25°C. Absorption measurements were made with a Jasco V-550 spectrophotometer with a 1 cm path length quartz cuvettes at a protein concentration of 6 µM. Near-UV and visible CD spectra were recorded in the presence of 20 μM PLP on a Jasco J-710 spectropolarimeter equipped with a thermostatically controlled compartment at 25°C by using 1 cm path-length quartz cuvettes at protein concentrations between 5 and 10 μM. Routinely, three spectra were recorded at a scan speed of 50 nm/min with a bandwidth of 2 nm and averaged automatically. For far-UV measurements, the protein concentration was 1 μM with a path length of 0.1 cm. Fluorescence measurements were made with a FP750 Jasco spectrofluorimeter at a protein concentration of 1 μM in the presence of 10 µM PLP. ANS emission spectra were registered upon excitation at 365 nm of a 1 μM enzyme sample previously incubated with 20 μM ANS for 1 h on ice in the presence of 10 µM PLP.

### Size-Exclusion Chromatography

SEC experiments were performed using an AktaPure FPLC system (GE Healthcare) and a Superdex 200 Increase 10/300 GL column equilibrated and run in 50 mM HEPES pH 8.0, containing 150 mM NaCl and 20 μM PLP. Each variant was dissolved in the running buffer at a concentration of 10 μM and incubated for 20 min at 25°C. Then, 0.1 ml of sample were loaded on the column and eluted at a flow rate of 0.5 ml/min. Chromatographic profiles were analyzed by the Unicorn v7.3 software (GE Healthcare).

### Limited Proteolysis

hOAT wild-type or the Q90E, R271K, E318K and C394Y variants were incubated with proteinase K at a 1:100 protease/enzyme ratio (w/w) at 25°C in PBS buffer pH 8.0 in the presence of 20 μM PLP. Aliquots were withdrawn at different times and treated with 2 mM PMSF. Five micrograms of each sample were loaded on a 12% SDS-PAGE gel.

### Cell Culture and Lysis

Hek293 cells knock-out for the OAT gene (Hek293-OAT_KO) cells were cultured in DMEM supplemented with 10% fetal bovine serum, glutamine (4 mM), penicillin (100 units/ml) and streptomycin (100 μg/ml) at 37°C in a humidified 5% CO_2_ environment. For lentiviral-based stable expression of hOAT forms, Hek293-OAT_KO cells were infected with lentiviral particles containing pLENTI6CMV-DEST vectors encoding each species and selected with 5 mg/ml hygromycin for construct integration into the genome.

To test the effect of PN, PL and PM, cells were cultured in Petri dishes in Ham’s F12 Glutamax medium in the absence or presence of 10 μM of each vitamer for 4 days. In each experiment, cells were harvested and lysed in CHAPS buffer (1% w/v CHAPS, 100 mM KCl, 20 mM HEPES, 1 mM EGTA), pH 8.0 plus protease inhibitor cocktail (Complete Mini, Roche) and 100 μM PLP for 30 min on ice. The whole cell extract was separated by centrifugation (29,200 g, 10 min, 4°C) to obtain the soluble fraction. The pellets were then resuspended in an equal volume of denaturing gel loading buffer to obtain the insoluble fraction. Protein concentration in the soluble cell lysate was measured using the Bradford assay.

### Blue Native Page

Mitochondrial isolation was carried out by sequential centrifugation. In brief, cultured cells were collected using a scraper and centrifuged for 10 min at 600 g in PBS. The pellet was homogenized using a Teflon pestle operated at 1,600 rpm on ice. The homogenate was centrifuged at 600 g for 10 min. The supernatant was centrifuged at 7,000 *g* for 10 min. The pellet was resuspended with 200 μl of ice-cold isolation buffer and subjected to a last centrifugation step for 10 min at 7,000 *g*. All centrifugation steps were carried out at 4°C. Buffer composition and other technical details can be found in Frezza C. *et al.* (Doimo et al., 2013). Mitochondrial pellets were suspended in an appropriate volume of Native Buffer (Invitrogen) with Digitonin or DDM (Sigma-Aldrich) to a final concentration of 1% (w/v) and membrane proteins were solubilized during 1 h incubation on ice. After a 20 min centrifugation at 16,000 g, the supernatant was collected and 5% G250 (Invitrogen) was added to obtain a final concentration equal to one-quarter that of the detergent. 100–150 μg of mitochondrial membrane proteins were applied and run on a 3–12% Bis-Tris gel (Invitrogen) and visualized using Coomassie staining.

### Western-Blot

10–20 μg of the soluble or insoluble fraction of the cell lysate were loaded on a 10% SDS-PAGE gel, transferred on a nitrocellulose membrane and immunoblotted with anti hOAT antibody (1:1,000, OriGene Technologies Inc.) and horseradish peroxidase-conjugated anti-IgG antibody (1:5,000). GAPDH (1:100, Cell Signaling Technology) antibody was used as marker protein for total and soluble extracts while porin (1:2,000, AbCam) was used as marker for mitochondrial extracts. Immunocomplexes were visualized by an enhanced chemiluminescence kit (ECL, Pierce Biotechnology, Rockford, IL). For cross-linking experiments 25 µg of the whole cellular lysate was treated with BS(PEG)_5_ at 1 mM or 10 mM final concentrations, and quenched in 0.5 M Tris-HCl pH 7.5 after 30 min 20 µg of each sample were analyzed by western blot. Measurement of protein half-lives by cycloheximide chase assays were performed as described in (Montioli et al., 2013). Briefly, Hek293-OAT_KO cells expressing each analyzed species were treated with cycloheximide (final concentration 10 mg/ml). At different times (0, 8, 12, 24 and 48 h) cells were harvested, lysed, and OAT levels were measured by western-blot, as described above.

### Immunofluorescence Microscopy

Hek293-OAT_KO cells expressing hOAT wild-type and the Q90E variant were seeded into a 24-well plate containing a 13 mm glass cover-slip and grown for 24 h at 37°C under O_2_/CO_2_(19:1). Cells were incubated with Mitotraker Red at 37°C for 30 min. Cells were fixed in methanol, permeabilized with 0.3% Triton X-100 in PBS and then blocked in 3% bovine serum albumin, 1% glycine in PBS. For immunolabeling, mouse anti-hOAT (Acris Antibodies, 1:50) was used as primary antibody, and Alexa Fluor conjugated antibodies (Life Technologies) were used as secondary antibodies. Nuclei were stained with Dapi, and the coverslips were mounted over slides in AF1 medium (Dako). Images were captured using a Zeiss Axio Observer Z1 equipped with Apotomeanddigital Camera AxiocamMRm (Zeiss). For figure preparation, images were processed using Adobe Photoshop.

## Results and Discussion

### Bioinformatic Analyses

To gain insights into the possible structural and/or functional effects of each amino acid substitution, we carried out a preliminary inspection of the microenvironment of each mutation site (Figure 1) followed by an in silico mutagenesis study (Supplementary Figure S1). Gln90 lies on the loop 75–95 and contacts the backbone of Ala293 and Asn89 by two hydrogen bonds (Figure 1B). The substitution of Gln90 with Glu probably changes the local conformation of the loop 75–95 since it would eliminate the possibility to form a hydrogen bond with Ala293 (Supplementary Figure S1A). Arg154 is located at the monomer/monomer interface of the dimeric unit and close to the dimer-dimer contact surface of the tetrameric unit of hOAT. It is in a proper position to contact Pro202 of the same subunit and both Pro199 and Phe197 of the neighboring subunit. The substitution Arg154-to-Leu is predicted to both abolish the aforementioned contacts and affect the conformation of the interface loop 195–203 (Supplementary Figure S1B). Gly237 belongs to the loop 229–245 containing Glu235. Crystallographic studies indicate that Glu235 could play a key role in guiding the ω-transaminase reaction toward L-Orn by shielding Arg413 from interacting with the α-carboxylate group (Storici et al., 1999). The in silico mutagenesis analysis (Supplementary Figure S1C), suggests that the G237D substitution could alter the 234–238 active site loop conformation, thus indirectly interfering with the position of Glu235. Arg271, located into a polar cleft, acts as a bridge between the N-terminal region and the C-terminal domain and its side chain interacts with both the Asn89 side chain and the Pro413 backbone oxygen (Figure 1B). The substitution Arg271-to-Lys probably makes the residue unable to support both interactions (Supplementary Figure S1D) leading to a possible impact on the tertiary structure. Glu318 is exposed on a surface region and interacts with the side chain of Lys155 through a salt bridge (Figure 1E). Such a contact is expected to be an important anchoring point for the loop 313–327, which goes through the active site of the neighboring subunit and contributes to PLP binding through Thr322 (Supplementary Figure S1E). The substituted amino acid, although is not predicted to give rise to remarkable alterations, could assume a different rotameric conformation or change the surface electrostatic potential. Lastly, Cys394 lies on the helix 390–400 of the C-terminal domain in a position suitable to contact the backbone oxygen of Ala404 by a hydrogen bond (Figure 1F). The substitution of Cys394-to-Tyr possibly abolishes the contact with Ala404 and introduces a steric hindrance between the C-terminal and the N-terminal domain. This is predicted to alter the conformation of the C-terminal loop 400–411 and of the N-terminal loop 50–63, both located at the entrance of the active site (Supplementary Figure S1F).

### Expression Studies of the Q90E, R154L, G237D, R271K, E318K, and C394Y Variants in *E. coli*

To provide insights into the structural and/or functional effect of the selected amino acid substitutions in hOAT, we inserted each mutation on the protein cDNA cloned in a prokaryotic expression vector by site-directed mutagenesis. We then compared expression level and specific activity of each variant with that of the wild-type in *E. coli* cultures. As shown in Supplementary Figure S2A, the selected variants showed different behaviors suggesting the presence of different defects. Two out of six mutations (Q90E, R271K) decreased at similar extents both specific activity and soluble protein levels in western-blot. Although the absence of a catalytic impairment cannot be ruled out, the data suggest that these variants are endowed with a folding defect that reduces protein levels by promoting aggregation and/or degradation. Both the E318K and the C394Y variants showed a reduction in specific activity more pronounced that the reduction in soluble protein levels, a finding that does not allow to rule out a catalytic defect along with a possible structural impairment. In the case of the E318K, soluble protein levels were unaltered and residual specific activity was 49%, in line with results in yeast expression systems evidencing that the E318K mutation caused mild effects in terms of hOAT expression and functionality (Doimo et al., 2013). Finally, the R154L and G237D mutations strongly affected hOAT specific activity, but the reduction was not accompanied by significant changes of the levels of soluble protein. Thus, it can be envisaged that the R154L and G237D variants are mainly affected by a catalytic defect. In line with these results, the R154L variant was found to produce normal amounts of expressed protein but undetectable enzymatic activity in patient fibroblasts and in transfected mammalian cells (Brody et al., 1992). Similarly, the hOAT enzymatic activity in cells of a patient bearing the G237D mutation resulted undetectable (Mashima et al., 1992; Ohkubo et al., 2005). In line with these results, a qualitative analysis of insoluble fraction of the bacterial lysate (Supplementary Figure S2B) shows an increased presence of high MW species only in cells expressing the Q90E, R271K, E318K, and C394Y variants, suggesting that these species could be more prone to misfolding, which in bacterial cells usually drives aggregation in inclusion bodies. Therefore, considering that the expression in *E. coli* offers the possibility to produce and purify sufficient quantities of the examined variants, although at different extents, we followed this approach to have a more deep knowledge of their structural and catalytic features.

### Biochemical Studies of the Purified Recombinant Q90E, R154L, G237D, R271K, E318K, and C394Y Variants

All variants were homogenous as indicated by a single band in SDS-PAGE. However, the G237D variant exhibited a mobility slightly slower with respect to the other species (Supplementary Figure S3A). Maldi-TOF mass spectrometry analysis of this variant yielded a molecular weight difference of 72.8 Da as compared with the wild-type (Supplementary Figure S4), a value compatible with the substitution of glycine with aspartic acid. Thus, this abnormal SDS-PAGE migration can be due to a mechanism of “gel shifting,” already observed for a set of cytosolic proteins (Shi et al., 2012). Moreover, the overall shape of the far-UV CD spectra of the variants were identical to that of wild-type hOAT indicating that the mutations do not affect the secondary structure of the protein Supplementary Figures S3B,C). To evaluate the impact of the examined mutations on catalytic activity, the steady-state kinetic parameters for the overall transamination of the L-Orn/αKG pair were measured and compared with those of wild-type hOAT (Table1). The Q90E, R271K, and E318K variants did not show biologically meaningful reductions of the *k*_cat_/K_m_ values, thus indicating that the mutation of Gln90, Arg271, or Glu318 does not cause a catalytic defect. A special mention should be made for the C394Y variant that displayed 1) an increased K_m_ for α-KG leading to a∼45-fold decrease of catalytic efficiency with respect to wild-type hOAT, and 2) a substrate inhibition kinetics in the presence of increasing L-Orn concentrations giving a K_i_ equal to 9.7 ± 1.4 mM (Supplementary Figure S5). The kinetic parameters of this variant have been determined using a minimal steady-state model related to the formation of an unproductive enzyme-substrate complex after the simultaneous binding of two or more substrate molecules at the active site. Nevertheless, the structural bases of this behaviour are currently unknown. Conversely, the R154L and G237D variants exhibited a dramatic decrease of catalytic activity with respect to the wild type. Their kinetic parameters were difficult to obtain because of the very slow reaction rate. In fact, when 3 µM R154L or G237D variant was allowed to react with 100 mM L-Orn and 100 mM α-KG, P5C was produced with an initial rate of about 0.1 and 0.08 s^−1^, respectively. Assuming that these values are close to their *k*_cat_ values of the transamination, the rate of the reaction of the R154L and G237D variants is about 350- and 437-fold lower, respectively, than that of the wild-type enzyme. These data are compatible with what suggested by the computational analyses.

**TABLE 1:** Kinetic parameters of the overall transamination of hOAT wild-type and variants. Experiments were performed in 50 mM HEPES buffer pH 8.0, 150 mM NaCl, at 25°C in the presence of saturating PLP.

Enzyme	Substrate	Co-substrate	*k*_*cat*_ (s^−1^)	K_M(L-Orn)_ (mM)	K_M(α-KG)_ (mM)	*k*_*cat*_/K_M_ (mM^−1^s^−1^)
WT	L-Orn	α-KG	34.9 ± 0.6	6.5 ± 0.4	—	5.4 ± 0.3
	α-KG	L-Orn	35.7 ± 0.7	—	3.9 ± 0.5	9.1 ± 1.2
Q90E	L-Orn	α-KG	21.3 ± 0.4	3.6 ± 0.3	—	5.9 ± 0.5
	α-KG	L-Orn	19.2 ± 0.3	—	5.6 ± 0.2	3.4 ± 0.1
R154L	L-Orn	α-KG	0.1 ± 0.01	nd	nd	nd
	α-KG	L-Orn				
G237D	L-Orn	α-KG	0.08 ± 0.01	nd	nd	nd
	α-KG	L-Orn				
R271K	L-Orn	α-KG	12.9 ± 0.2	5.0 ± 0.3	—	2.6 ± 0.2
	α-KG	L-Orn	13.9 ± 0.2	—	6.5 ± 0.4	2.1 ± 0.1
E318K	L-Orn	α-KG	26.7 ± 1.4	4.4 ± 1.0	—	6.1 ± 1.4
	α-KG	L-Orn	26.7 ± 0.5	—	7.3 ± 0.5	3.7 ± 0.3
C394Y	L-Orn	α-KG	5.8 ± 0.4^a^	1.6 ± 0.2^a^	—	3.6 ± 0.6^a^
	α-KG	L-Orn	5.8 ± 0.4	—	28.8 ± 2.4	0.20 ± 0.02

a Data fitted to a non-hyperbolic curve taking into account substrate inhibition (K_i_ = 9.7 ± 1.4 mM).

**FIGURE 2:**
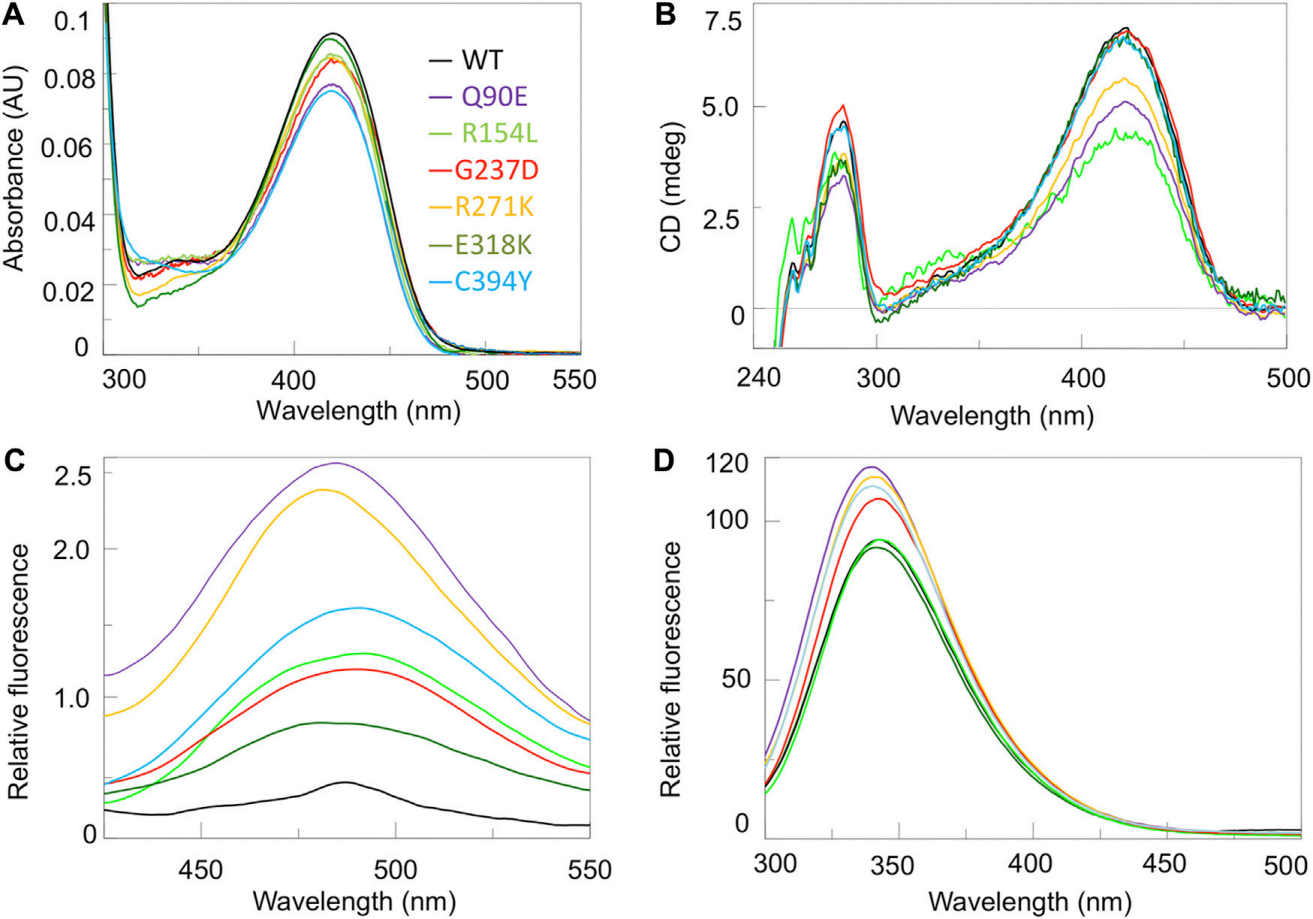
Spectroscopic characterization of hOAT variants. Absorbance **(A)**, UV-Vis CD **(B)**, ANS fluorescence emission **(C)** and intrinsic fluorescence emission spectra **(D)** of hOAT wild-type and the variants under study. The color code for all panels is reported as inset of panel A. Absorbance and CD spectra were registered at 6 μM enzyme concentration while ANS and intrinsic fluorecence were measured at 1 μM enzyme. CD and fluorescence analyses were performed in the presence of exogeouns PLP. All measurements were performed in 50 mM HEPES pH 8.0, 150 mM NaCl.

As in all PLP-enzymes, the coenzyme is bound through a Shiff base with a lysine residue of the apoprotein, a complex called internal aldimine that generates typical absorbance bands in the visible region (Liang et al., 2019). Coenzyme binding to hOAT gives rise to an absorbance band at 420 nm and a shoulder at 340 nm associated with positive dichroic signals, previously attributed to the ketoenaminic and enoliminic tautomeric forms of the Lys292 internal aldimine, respectively (Shen et al., 1998; Montioli et al., 2017). As shown in Figures 2A,B, the variants exhibited visible absorbance and CD spectra qualitatively similar to those of the wild-type, although in several cases they displayed a lower intensity of the absorbance and/or dichroic signal. Their optical activity values (Supplementary Table S3) were also comparable to that of 76 millidegree/A_422nm_ of wild-type hOAT, thus suggesting that the mutations do not significantly alter the microenvironment of the internal aldimine. The only exception was the R154L variant which exhibited an optical activity value ∼1.6-fold lower than that of the wild-type enzyme indicating that in this variant the chiral microenvironment of the cofactor is altered.

We acquired the near-UV CD (Figure 2B), as well as ANS and intrinsic fluorescence emission (Figures 2C,D) spectra of the enzymatic species under study. Near-UV and intrinsic fluorescence spectra provide insights into possible changes of the tertiary structure of hOAT, while ANS emission spectra give information about changes of hydrophobic surfaces exposed to the solvent. Taking into account the magnitude of the dichroic signal, the emission intensity, and the maximum of the fluorescence spectra of each variant with respect to the wild-type, it can be inferred that changes consisting in a remarkable alteration of the microenvironment of aromatic amino acids and an increased exposure of hydrophobic surfaces are present on Q90E and R271K, while they are appreciable at a different degree for the remaining variants. The E318K species shows the less pronounced alterations only consisting in a slight increase in the ANS signal, while more evident spectral changes are observed for the R154L, G237D and C394Y variants, suggesting a structural defect. However, considering that these species also show active site alterations translating into a catalytic defect, it is not possible to establish if and to what extent the observed changes in tertiary structure represent conformational changes in the microenvironment of the active site. The quaternary structure was also analyzed by SEC. All variants, except the R154L, displayed a tetrameric assembly as previously observed for the wild-type (Montioli et al., 2017) (Supplementary Figure S6). In fact, the R154L variant exhibited an elution volume corresponding to that of the artificial dimeric variant R217A (Montioli et al., 2017) indicating that the R154L mutation affects hOAT tetramerization. The proximity of the mutation site to the tetramer interface highlighted by in silico analyses, could explain the perturbation of the tetramer-dimer equilibrium. It should be noticed that the different quaternary structure of the R154L variant does not probably relate to its kinetic defect, considering that the tetrameric assembly is not crucial for hOAT catalysis (Montioli et al., 2017).

Taken together, these data indicate that 1) the R154L and G237D variants display a predominant catalytic defect, in line with the *E. coli* expression studies showing a strongly reduced specific activity not paralleled by reduced protein levels; 2) the Q90E and R271K variants exhibit a folding defect more pronounced than a catalytic one. It should be noted that the residues Gln90 and Arg271 are close to each other at the interface between the N-terminal and large domain. The finding that their mutation leads to the most prounced alterations in the hOAT tertiary structure is in line with the low protein levels upon expression in *E. coli*, suggesting a reduced efficiency of the folding process probably driven by a conformational change of the loop 75–95 (Supplementary Figures S1A,D); 3) the E318K and C394Y variants show a complex behavior. When expressed in bacteria, the E318K shows a reduction of specific activity that could be partly ascribed to the 23% reduction in *k*_cat_, and partly to a conformational defect possibly promoting the formation of insoluble aggregated species. In this regard, it is noteworthy that, despite the high expression level in the soluble fraction of the *E. coli* lysate, the purification yield of E318K resulted considerable lower than those of the wild-type, R154L and G237D (*Expression and Purification of Mutants*). This suggests that E318K could partly undergoes aggregation or degradation during the purification procedure. The C394Y appears to be affected by a structural defect more pronounced than the E318K, in line with a more remarkable reduction of protein levels in the bacterial lysate, accompanied by a catalytic defect.

A possible conformational change caused by the Q90E, R271K, E318K, and C394Y mutations in hOAT was corroborated by limited proteolysis experiments. As shown in Supplementary Figure S7, although all species exhibited a similar proteolysis pattern, the variants were digested with different rates as compared to the wild-type. In particular, wild-type hOAT underwent the removing of a short amino acid stretch generating a product with an apparent MW of 43 KDa, which did not undergo any further degradation up to 50 min. A qualitatively similar behavior was observed for the R271K, E318K and C394Y variants, although the cleavage occurred with a more rapid kinetics with respect to wild-type hOAT. A different trend was observed for the Q90E variant, which clearly exhibited a progressive degradation of both the native and the cleaved species. These data agree with those obtained in bacterial lysates and/or purified proteins suggesting that the Q90E, R271K, E318K, and C394Y variants are affected by a structural alteration, which is more pronounced for the Q90E as compared to the other variants. This change could possibly translate into an increased degradation propensity in a eukariotic cellular environment (see below). Therefore, to identify the possible cellular relapse of the folding defect, we decided to explore the behavior of the Q90E, R271K, E318K, and C394Y variants in a model base on cells of human origin.

### Expression of Q90E, R271K, E318K, and C394Y Variants in a Cellular Model of GA

**FIGURE 3:**
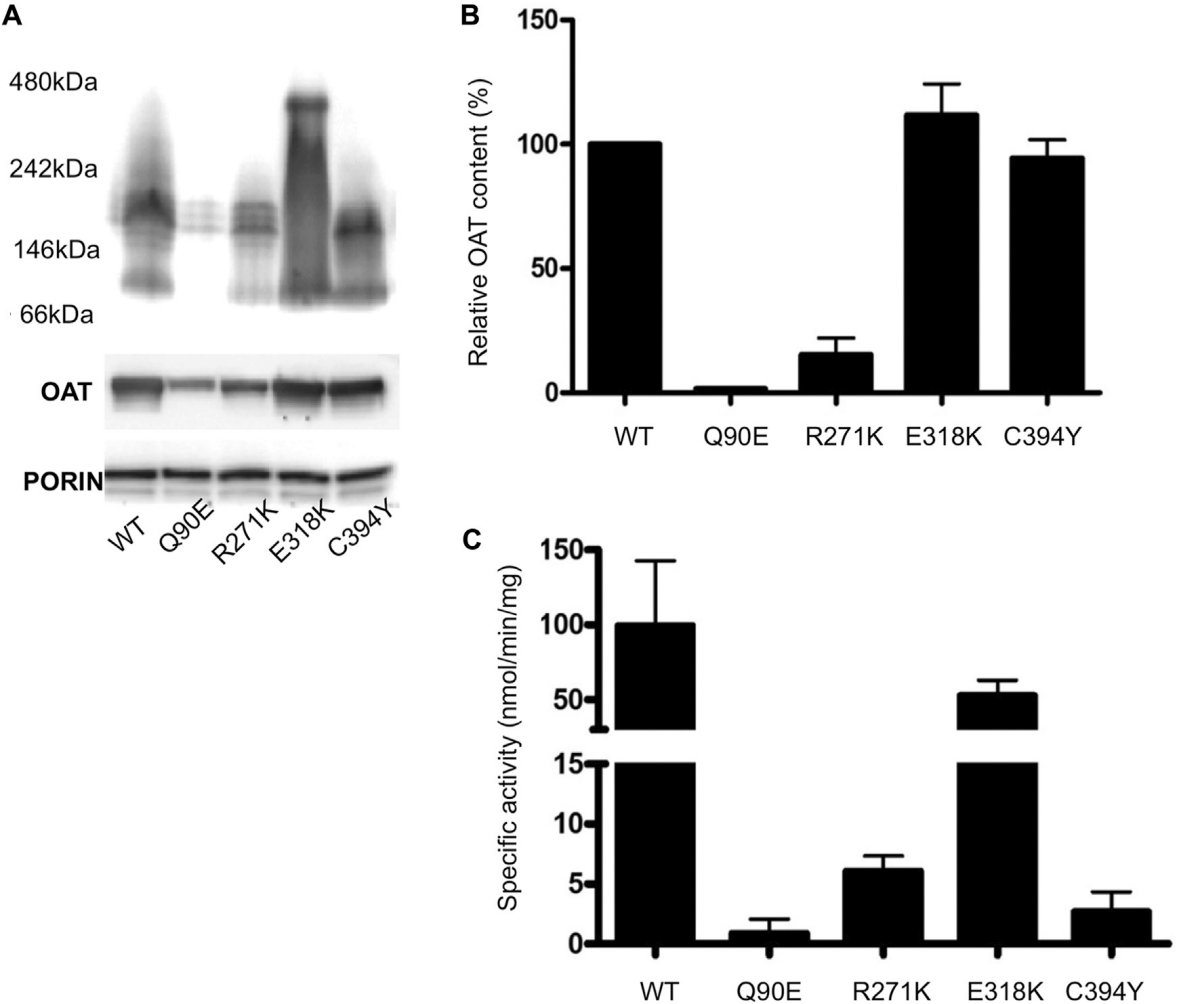
Analysis of hOAT variants expressed in Hek293-OAT_KO cells cultured in DMEM medium. **(A)** BN-PAGE. Purified mitochondria were solubilized in the presence of 1% DDM, centrifuged at 16,000 g for 20 min. The soluble fractions were run on a 3–12% Blue Native gel. The total OAT content of each sample under denaturing conditions is also shown. An antibody against porin was used as loading control. **(B)** Relative OAT content evaluated by ImageJ sofware. **(C)** Transaminase activity. 100 μg of soluble lysate was incubated with 100 mM L-Orn, 10 mM α-KG, and 200 μM PLP at 25°C in 50 mM HEPES buffer, pH 8, 150 mM NaCl. The absorbance of pyrroline-5 carboxylate derivatized with aminobenzaldehyde was measured at 440 nm. Data represent the mean fo at least three independent experiments.

We analyzed the behavior of the variants endowed with a structural defect in a cellular model of GA based on Hek293-OAT_KO cells (Montioli et al., 2018) stably expressing each species by lentiviral infection. As shown in Figure 3, in a high B6 medium (DMEM) Q90E and R271K variants displayed specific activity and expression levels reduced to less than 2% and 7–15%, respectively, as compared to wild-type hOAT, and a tetrameric assembly in BN-PAGE, in line with the results obtained with purified proteins. The E318K variant showed protein levels in SDS-PAGE similar to those of the wild-type, and a smear of oligomers and high-order aggregates in BN-PAGE, with no defined bands relatable to tetrameric species. Nevertheless, its specific activity was only reduced by 27%, probably because of the increased stability of the oligomers and aggregates in the assay mixture, where a lower concentration and excess of substrate and coenzyme is present, as compared with the BN-PAGE mixture. Finally, the C394Y variant showed oligomeric assembly and protein levels similar to those of the wild-type, but its specific activity was reduced to 3%, thus confirming the occurrence of a functional alteration. The correlation between a reduced specific activity and reduced protein levels or altered assembly for the Q90E, R271K and E318K variants allows to envisage a folding defect, in agreement with *E. coli* expression studies. On the other hand, in the case of the C394Y variant we observed a strongly reduced specific activity in line with its catalytic defect, but we did not detect variations in proteins levels as compared with wild-type hOAT. The latter feature is different from what observed upon expression in *E. coli*. It can be speculated that the expression levels of Hek293 cells are low enough to promote the folding of the variant in a enriched culture medium through the action of molecular chaperones.

**FIGURE 4:**
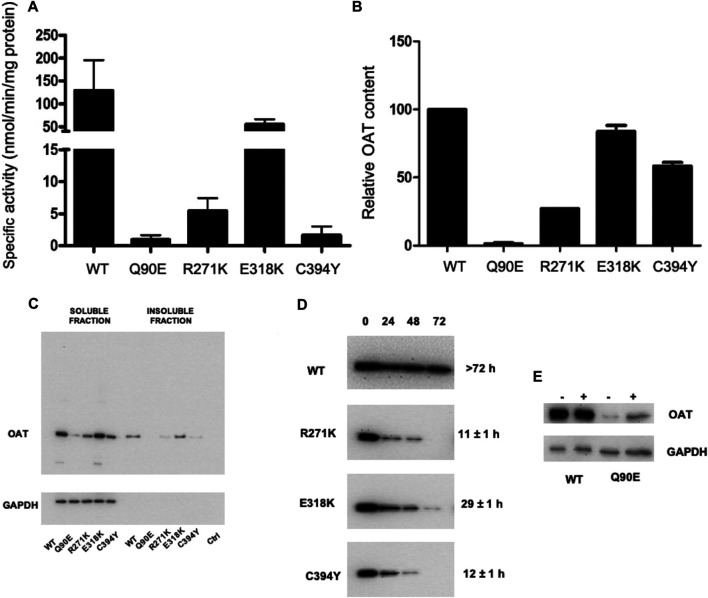
Analysis of hOAT variants expressed in Hek293-OAT_KO cells cultured in Ham’s F12 medium. Hek293-OAT_KO cells expressing each species were growth for 4 days in Ham’s F12 medium and lysed in CHAPS buffer. **(A)** Transaminase activity. 100 μg of soluble lysate was incubated with 100 mM L-Orn, 10 mM α-KG, and 200 μM PLP at 25°C in 50 mM HEPES buffer, pH 8, 150 mM NaCl. The absorbance of pyrroline-5 carboxylate derivatized with aminobenzaldehyde was measured at 440 nm. Data are expressed as mean ± S.E.M. (*n* = 3). **(B)** 10 μg of the soluble lysate were subjected to SDS-PAGE, immunoblotted with anti-OAT from mouse (1:1,000), and detected by chemiluminescence. Data are expressed as mean ± S.E.M. (*n* = 3). **(C)** Representative western-blot analysis of 10 μg of total protein levels in the soluble and insoluble fraction. **(D)** Half-life determination by cycloheximide assay. The image shows representative western-blot analyses. For each sample, the relative intensity with respect to that at the start of the chase was used to calculate the half-life. Data are the mean of two independent experiments. **(E)** Changes in Q90E expression levels upon treatment with 10 μM MG132.

To gain deeper insights into the behavior of each variant, we studied their properties upon culturing cells in a medium containing a B6 concentration of the same order of magnitude than that of biological fluids (Ham’s F12) (Figures 4A,B). In agreement with the data obtained in DMEM, Q90E and R271K displayed specific activity and expression levels reduced by 125-fold and 18-fold, respectively, as compared with wild-type hOAT. In the case of Q90E, the expression was so low that we detected only a faint band in western-blot (Figures 4A–C). E318K showed expression level and specific activity similar to those of wild-type hOAT. Finally, C394Y displayed a strong reduction of specific activity that is not paired by a remarkably reduced expression level. Interestingly, by determining the kinetic parameters of the transamination reaction in lysates of cells expressing the C394Y variant (Supplementary Figure S5), we confirmed the occurrence of a substrate inhibition phenomenon in line with the demonstrated functional defect of the purified variant. Notably, at the L-Orn concentrations present in GA patients, the C394Y variant could be inhibited, a mechanism possibly contributing to pathogenesis.

By a deeper investigation of the intracellular behavior of wild-type hOAT in Ham’s F12 medium (Figure 4C **)** we found that 1) more than 90% of the wild-type was present in the soluble fraction; 2) the reduced protein levels of the Q90E and R271K variants were not due to an increased amount of insoluble protein; 3) the E318K variant showed subtle differences with respect to the wild-type consisting in a barely detectable increase in the relative amount of protein present in the insoluble fraction, the appearance of aggregates in western-blot upon long exposure (data not shown), and the presence of a slightly higher proportion of high MW species in cross-linking experiments that was not possible to quantify (Supplementary Figure S8). In addition, wild-type hOAT was endowed with a high intracellular stability, as shown by its half-life higher than 72 h similar to that reported for other mitochondrial proteins (Stotland and Gottlieb, 2015). On the other hand, in line with the limited proteolysis experiments on purified proteins, the R271K, E318K, and C394Y variants showed a reduced intracellular half-life (Figure 4D), being the R271K and the C394Y the most prone to be degraded. Mitochondria have a specific set of quality control systems, made up of proteases and molecular chaperones, which handle both internally-produced and nuclear-encoded proteins (Voos et al., 2016). Damaged proteins are targeted for degradation inside mitochondria, or, if homeostasis cannot be restored, through removal of damaged mitochondria by mitophagy (Stotland and Gottlieb, 2015). Thus, pathogenic hOAT variants showing conformational alterations could be more prone to be recognized by the mitochondrial quality control systems and to be directed to degradation. Although the reduction of half-life cannot be taken as a parameter to quantify the degree of conformational alteration of a variant, it should be noted that the E318K is the most stable. This is in line with clinical data on homozygous or compound heterozygous GA patients bearing the E318K mutation, where it has been observed a mild phenotype, with some indications of gene dosage effects (Mashima et al., 1999).

As for the Q90E variant, we could not determine its half-life due to the low expression levels. Nevertheless, we obtained indirect evidence for its propensity to proteolytic degradation by the finding that treating cells expressing the variant with MG132 caused a significant increase in protein levels (Figure 4E). Although the turnover of some mitochondrial precursor proteins occurs before reaching the mitochondrial compartment (Radke et al., 2008; Bragoszewski et al., 2013), MG132 is also able to inhibit mitochondrial proteolytic enzymes (Smith and Schnellmann, 2012; Ajit Bolar et al., 2013). Thus, the degradation inside mitochondria is the most probable explanation of the data. Nonetheless, since the Gln90 mutation could prevent hOAT mitochondrial import, we performed immunofluorescence microscopy experiments to define the subcellular localization of Q90E. These experiments confirmed the very low expression levels of the variant, but showed that it correctly localizes inside mitochondria (Supplementary Figure S9), as also indicated by the presence of a band in western-blot corresponding to the mature protein. In this regard, Kobayashi et al. (1995) have reported experiments in insect cells where the Q90E variant was present as a 49 kDa precursor and was not imported to mitochondria. This discrepancy can be understood considering that the human and insect mitochondrial import machinery and quality control systems are different, and that insect cells give rise to very high protein expression levels (Yee et al., 2018). Indeed, the same authors did not find the precursor band when the variant is expressed in mammalian cells (Kobayashi et al., 1995). Therefore, it cannot be excluded that the Gln90 mutation could interfere with the import kinetics and promote the degradation of the variant in the cytosol. A similar process has been already observed for a pathogenic form of the cytochrome c oxidase assembly factor 7 (Mohanraj et al., 2019).

Altogether, the picture coming from the combination of protein and cell studies confirms that the folding-defective pathogenic variants display detectable deviations from the behavior of hOAT wild-type and that the changes are less pronounced in a variant associated with a mild phenotype, the E318K, thus supporting the reliability of our experimental model.

### Effect of B6 Vitamers Treatment on Q90E, R271K, E318K, and C394Y Variants

One of the treatment options available to GA patients is the administration of Vitamin B6, which aims at increasing the intracellular concentration of PLP (Ramesh et al., 1988; Kennaway et al., 1989; Heller et al., 2017). It has been suggested that responsive patients bear mutations that preserve hOAT activity, and that the coenzyme could stabilize the protein by promoting holoenzyme formation (Kennaway et al., 1980; Ramesh et al., 1988; Kennaway et al., 1989). The analysis of the behavior of the wild-type enzyme has shown that in the absence of PLP it displays a less stable tetrameric assembly and is more prone to undergo aggregation under physiological conditions (Montioli et al., 2017). On these bases, the possibility that PLP could act as a chaperone for hOAT, as already observed for other disorders involving PLP-enzymes (Oppici et al., 2016), can be raised.

**FIGURE 5:**
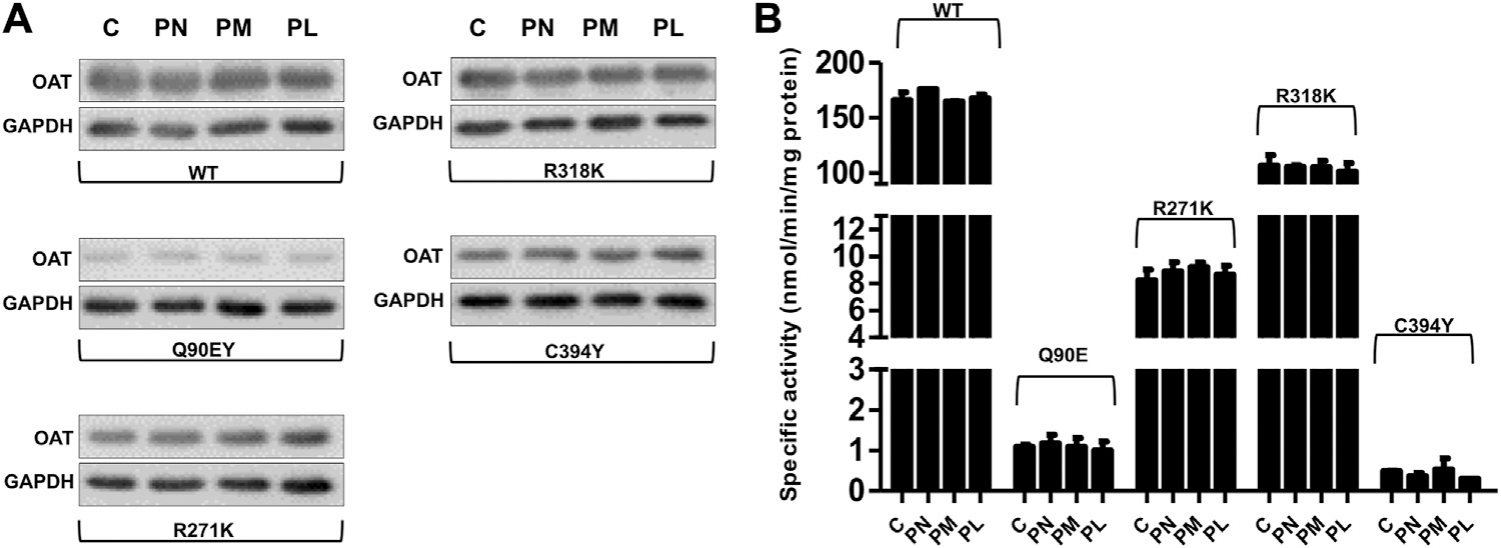
Effect of vitamers B6 on expression level and specific activity of OAT species in Hek293-OAT_KO cells. Hek293-OAT_KO cells overexpressing the indicated hOAT variants were growth for 7 days in Ham’s F12 medium in the absence (−) or presence (+) of the indicated vitamers B6 at 10 μM concentration. **(A)** 20 μg of the soluble lysate were subjected to SDS-PAGE, immunoblotted with anti-OAT from mouse (1:1,000), and detected by chemiluminescence. The images come from a single Western-blot and are representative of at least two independent experiments. **(B)** Transaminase activity was detected by incubating 100 μg of soluble lysate with 100 mM L-Orn, 10 mM α-KG, and 200 μM PLP at 25°C in 50 mM HEPES buffer, pH 8, 150 mM NaCl. Bar graphs represent mean ± S.E.M. of four independent experiments. C stands for the untreated control.

We tested the responsiveness of the Q90E, R271K, E318K, and C394Y variants by comparing their expression level and specific activity upon culturing cells for 1 week (a time sufficient to reach equilibrium in preliminary time course experiments) in Ham’s F12 medium in the absence or presence of vitamin B6. In particular we tested both the vitamers used in clinics, pyridoxine (PN), and the other two present in nature, pyridoxamine (PM) and pyridoxal (PL) (Albersen et al., 2014), all at 10 μM concentration. As shown in Figure 5A, B6 vitamers did not affect the overall protein expression levels and specific activity of hOAT wild-type and of the pathogenic variants. In this regard, although we could observe a slight increase in the specific activity of Q90E in the presence of PN and of R271K in the presence of PN or PM, the differences did not reach statistical significance (Figure 5B). This result was surprising, since although the clinical responsiveness of GA patients bearing the Q90E mutation is presently unknown, three patients bearing the E318K were found to be responsive (Mashima et al., 1999).

Thus, in our cellular model of GA, folding-defective pathogenic variants seem insensitive to coenzyme administration, apparently in contrast with clinical data. A discrepancy between clinical and *in vitro* data has been also observed in studies performed using yeast expression systems or patient fibroblasts (Doimo et al., 2013), and it merits some considerations. First, it should be mentioned that we could not adapt Hek293-OAT_KO cells for growing in a B6-free medium, which would have possibly allowed to maximise the effect of vitamin B6 addition, because of a low cell viability in the absence of PN. Second, all utilized *in vitro* models do not recapitulate the physiology of the retinal pigment epithelium, where the hOAT deficit gives rise to clinically-relevant changes. Thus, it can be hypothesized that human cell lines or yeast are suitable to analyze the consequences of missense mutations on the hOAT intracellular behavior, but are not sensitive enough to detect changes caused by coenzyme administration. However, it cannot be excluded that the B6-responsiveness of GA patients could be also due to molecular mechanisms independent of hOAT, such as to the promotion of alternative pathways of L-Orn degradation dependent on PLP. In this regard, studies performed on other mitochondrial PLP-enzymes in the purified form have evidenced that their folding is not promoted by the coenzyme (West and Price, 1990). By comparing the refolding of the cytosolic and mitochondrial isoenzymes of aspartate aminotransferase, it has been evidenced that, notwithstanding their high levels of sequence identity and structural similarity, they show an opposite behavior in refolding studies. While the cytosolic enzyme shows reversible unfolding in the presence of PLP, the mitochondrial counterpart is not able to refold even in the presence of the coenzyme (West and Price, 1990). Needless to say, more detailed studies on both the folding process of purified hOAT and the role of the coenzyme are necessary to shed light on this issue.

## Conclusion

In rare diseases associated with the malfunction of a protein, any pathogenic variant might show alteration of different functional or structural properties. This complicates the analysis of the underlying origin of diseases and, as a consequence, impairs a specific therapeutic approach. This is true in GA, where the detailed functional/structural effect(s) of most disease-causing mutations at protein level, i.e., the enzymatic phenotype, are presently unknown, with the exception of the V332M and R180T variants. Here, we tried to expand the spectrum of the enzymatic phenotypes leading to the hOAT deficit by defining the effects of representative missense mutations identified in homozygous patients, and involving residues located in different hOAT domains. Our data indicate that 1) the R154L and G237D mutations give rise to a remarkable loss of catalytic activity, which is probably the main responsible for pathogenicity; 2) the Q90E and R271K mutations cause a folding defect leading to a reduced intracellular half-life, which leads to a hOAT deficit because it reduces the amounts of functional protein; 3) the E318K and C394Y variants are endowed with a combination of functional and structural defects. The E318K mutation gives rise to mild kinetic alterations and a slight folding defect that mainly translates into a reduced hOAT half-life, in line with the milder phenotype of GA patients bearing this mutation (Mashima et al., 1999). In the C394Y variant, the folding defect is also accompanied by a functional alteration consisting into a substrate inhibition phenomenon that possibly contributes to pathogenicity. In contrast with clinical data, even if in limited number, we also found that Vitamin B6 is not able to induce a significant rescuing effect in none of the GA-associated mutations causing folding defects in hOAT. The latter finding opens the question on the distinction in B6-responsive and non-responsive GA patients (Montioli et al., 2021) and the search of other approaches aiming at counteracting misfolding for hOAT variants showing folding defects. Overall, these results allow us to expand the understanding of the molecular basis underlying GA pathogenesis as necessary premise for the establishment of genotype/phenotype correlations, an important gap of knowledge for this disorder.

## Data Availability

The original contributions presented in the study are included in the article/supplementary material, further inquiries can be directed to the corresponding authors.

## Abbreviations

hOAT, human ornithine aminotransferase; GA, gyrate atrophy; L-Orn, l-ornithine; αKG, α-ketoglutarate; P5C, pirroline-5-carboxylate; PLP, pyridoxal 5′-phosphate; PN, pyridoxine; PM, pyridoxamine; PL, pyridoxal; ANS, 1,8-anilino-naphtalene sulfonic acid; Hek293-OAT_KO, Hek293 cells knock-out for the OAT gene; BS (PEG) 5, PEGylated bis (sulfosuccinimidyl) suberate; CD, circular dichroism; WT, wild-type.
