# Supplementary material for: Molecular and Cellular Studies Reveal Folding Defects of Human Ornithine Aminotransferase Variants Associated With Gyrate Atrophy of the Choroid and Retina
Source: Front Mol Biosci. 2021 Jul 30;8:695205. doi: 10.3389/fmolb.2021.695205 (PMC8360850; doi:10.3389/fmolb.2021.695205)
Supplement: Supplementary file 1 [file DataSheet1.PDF]

SUPPLEMENTARY INFORMATION

Table S1. OAT variants expressed by homozygous GA patients and localization of the mutated residues in the hOAT crystal structure

OAT pathogenic variants	Structural region
Y55H, Q90E, G91E	N-terminal region
Q104R, A226V, Q233R, G237D, G121D, G142E, T181M, R154L, R180T, P199Q, P241L, R250P, T267I, A270P, R271K, H319Y, V332M, E318K, G353D	Large domain
G373E, C394Y, L402P, I436N,	Small C-terminal domain

Table S2. Sequence of primers used for site-directed mutagenesis. Mutated codons are underlined.

Primer name	Sequence (5'-3')
Q90E	CTTACAGTGCTGTCAACGAAGGGCATTGTCACC
R154L	CTGCCTGTAAACTAGCTCTTAAGTGGGGCTATACC
G237D	CAATTCAGGGTGAAGCAGACGTTGTTGTTCCGGATC
R271K	CAGACAGGATTGGCCAAAACTGGTAGATGGCTG
E318K	CATTAAGCCAGGGAAGCATGGGTCCACATACG
C394Y	GATGCTTGGAAGGTGTATCTACGACTTCGAGATAATG

Table S3. Optical activity values of hOAT wild-type and pathogenic variants

Enzyme	Optical activity
WT	76.6
Q90E	67.1
R154L	48.9
G237D	77.9
R271K	68.6
E318K	75.8
C394Y	71.2

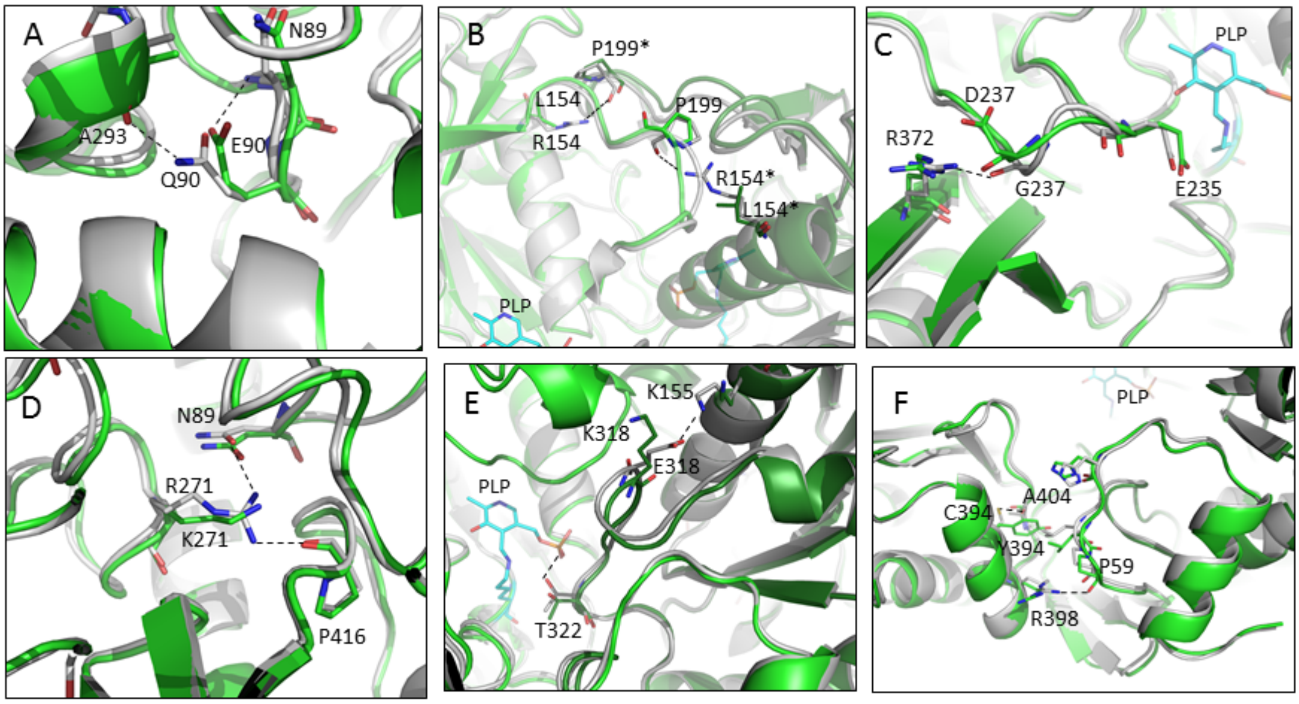

Fig. S1. *In silico* mutagensis of hOAT variants. Superimposition of wild type hOAT structure (PDB file 1OAT) and (A) Q90E, (B) R154L, (C) G237D, (D) R271K, (E) E318K and (F) C394Y structures obtained by *in silico* mutagenesis and energy minimization processes. Wild type and mutant structures are represented as gray and green ribbons, respectively. The two monomers are distinguished by light and dark colors. Amino acids in the environment of each mutation site are indicated and represented as sticks. PLP molecules are highlighted as cyan sticks. Images were rendered by the PyMol software (Schrödinger).

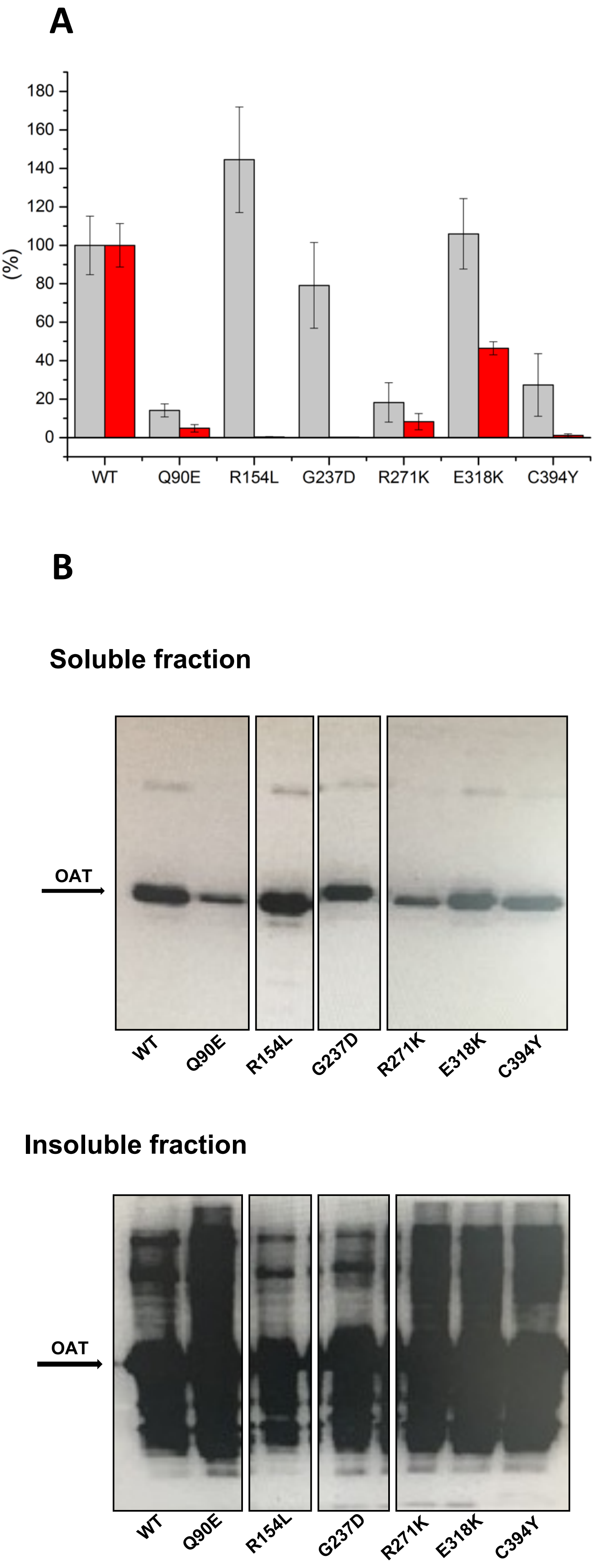

Fig. S2. Expression level and specific activity of hOAT variants upon expression in *E. coli*. A) Soluble protein levels (grey bars) and specific activity (red bars) of the indicated variants in crude lysates of *E. coli* cells transformed with the pOAT constructs encoding each species under study. The data are the mean ± S.E.M. of two independent experiments. B) Representative western blot of the protein content in the soluble and insoluble fraction of the bacterial lysate. *E. coli* cells expressing the indicated variants were harvested and lysed, 15 µg of soluble or insoluble fraction, as indicated, were subjected to SDS-PAGE, immunoblotted with anti-OAT from mouse (1:1000), and detected by chemiluminescence. The images come from a single Western-blot and are representative of two independent experiments.

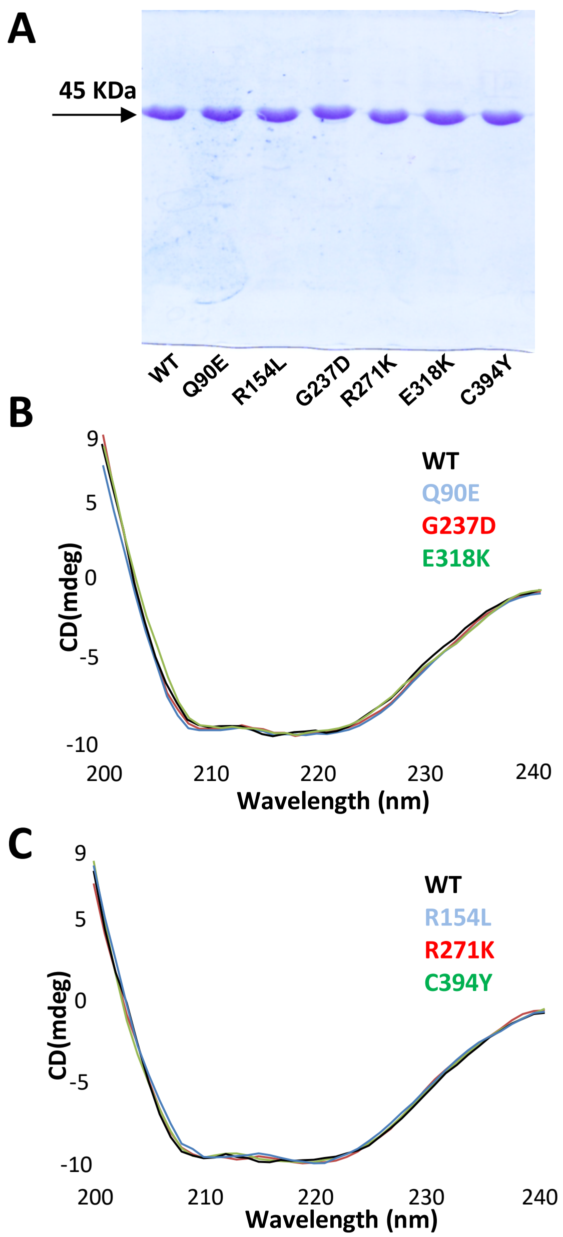

Fig. S3. SDS-PAGE analysis and Far-UV CD spectra of the purified variants. A) SDS-PAGE. After thermal denaturation for 5 min at 95°C in electrophoresis sample buffer, samples containing 4 µg of purified protein were loaded on a 12% SDS polyacrylamide gel and stained with Coomassie dye. B) Far-UV CD. Spectra were recorded at 1 µM enzyme concentration in HEPES 5 mM pH 8.0, 15 mM NaCl, in the presence of 10 µM PLP.

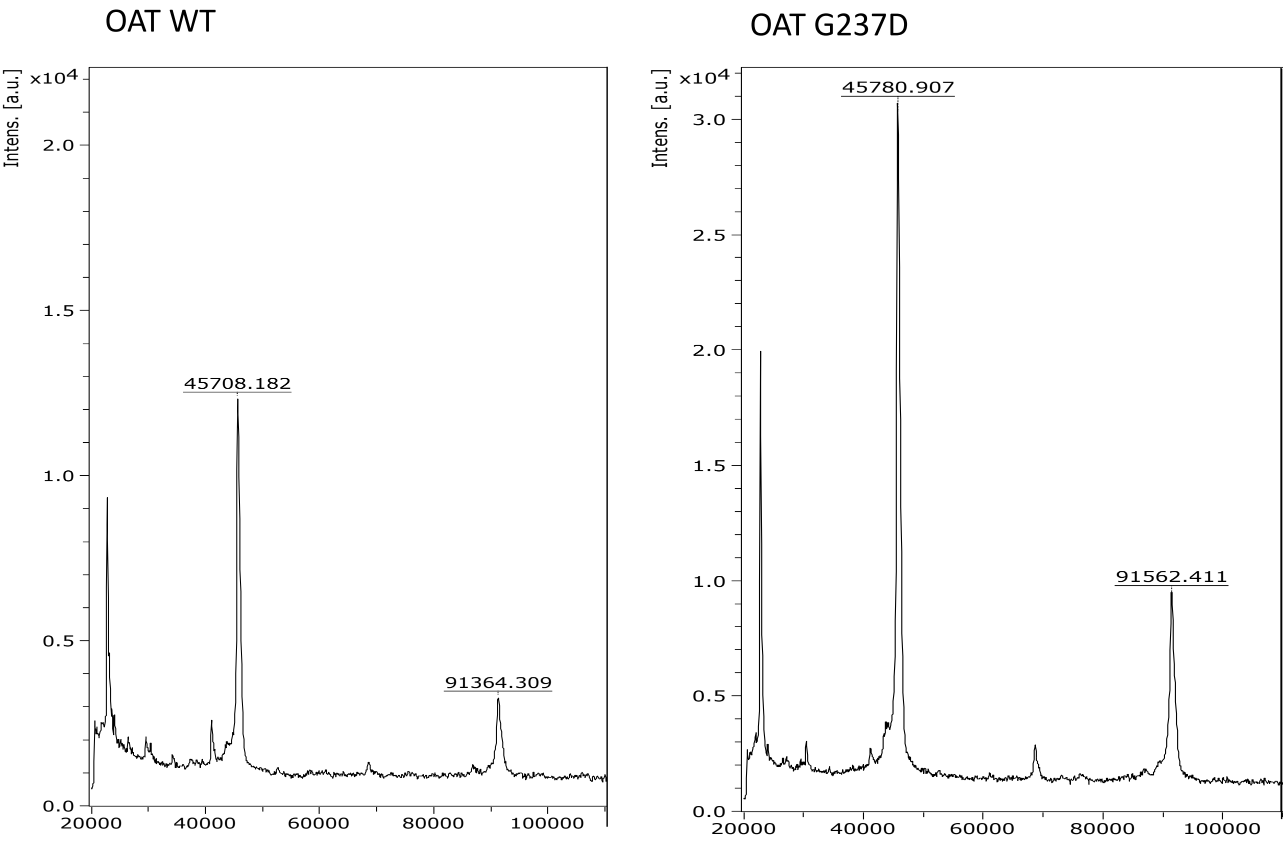

Figure S4. Maldi TOF MS analysis of wild-type hOAT and the G237D variant. hOAT wild type and G237D solutions at 100 µM concentration were mixed 1:1 (v/v) with the matrix solution (see M&M). 1 uL of each mixture was spotted in triplicate on to a Ground steel MALDI target plate (Bruker Daltonics), and allowed to dry at room temperature. Mass spectra were collected from m/z 60000 to 200000 in positive linear mode on a Bruker Ultraflextreme MALDI-TOF/TOF instrument (Bruker Daltonics).

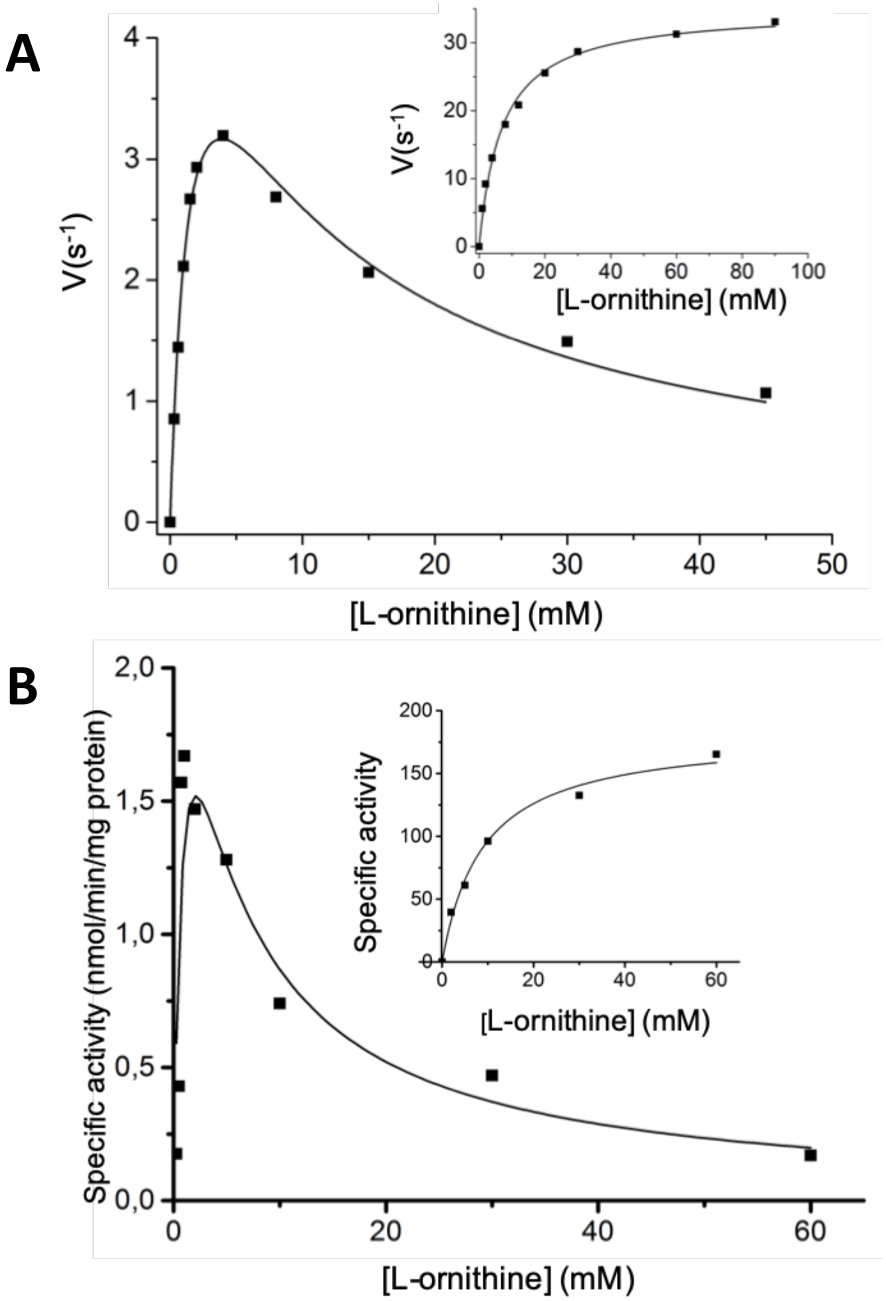

Fig. S5. Kinetic parameters of the C394Y variant. Plot of transamination rate *vs* L-Orn concentration of A) the purified C394Y variant and B) the cellular lysate of Hek293-OAT_KO cells expressing the C394Y variant, in comparison with the corresponding ones of wild type hOAT under the same experimental conditions (Inset of panels A and B). Plots and fittings were obtained using Origin 9.1 software.

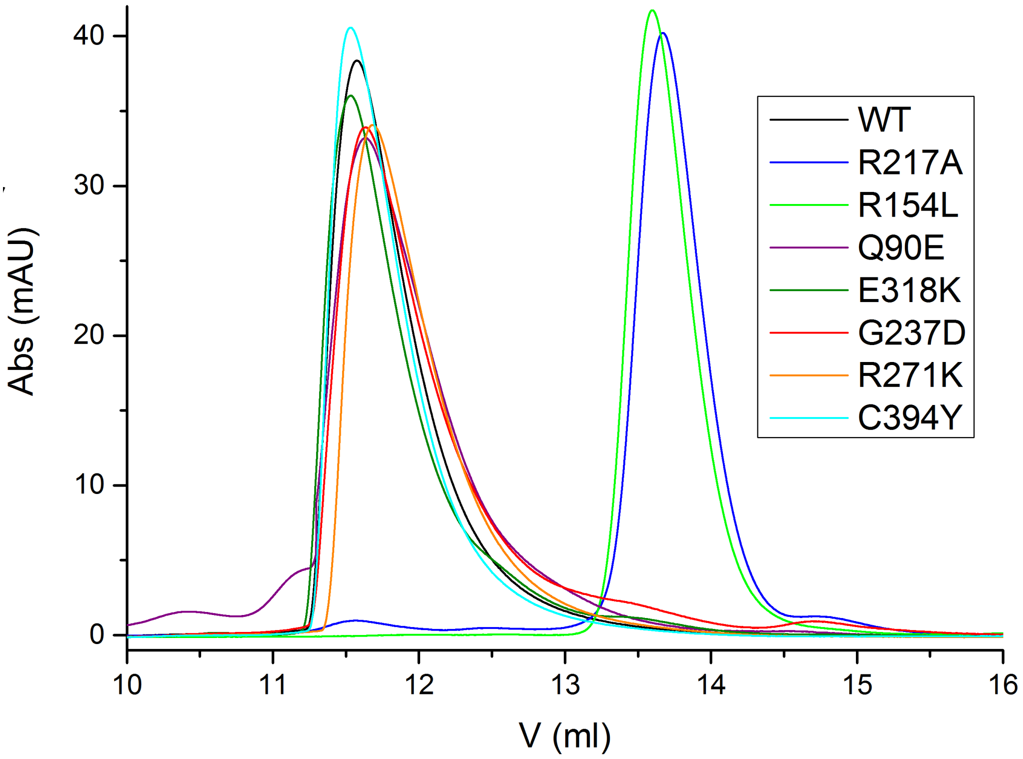

Fig. S6. SEC analysis of wild-type hOAT and variant. Each species was incubated in running buffer (HEPES pH 8.0, 150 mM NaCl, 20 µM PLP) at 10 µM concentration and loaded on a Superdex 200 increase 10/300 column. The color code of the chromatographic profiles is reported in the inset. Please note that the small peaks corresponding to higher order oligomers present in the elution profile of Q90E are probably due to the remarkable instability of this variant that generates aggregated species.

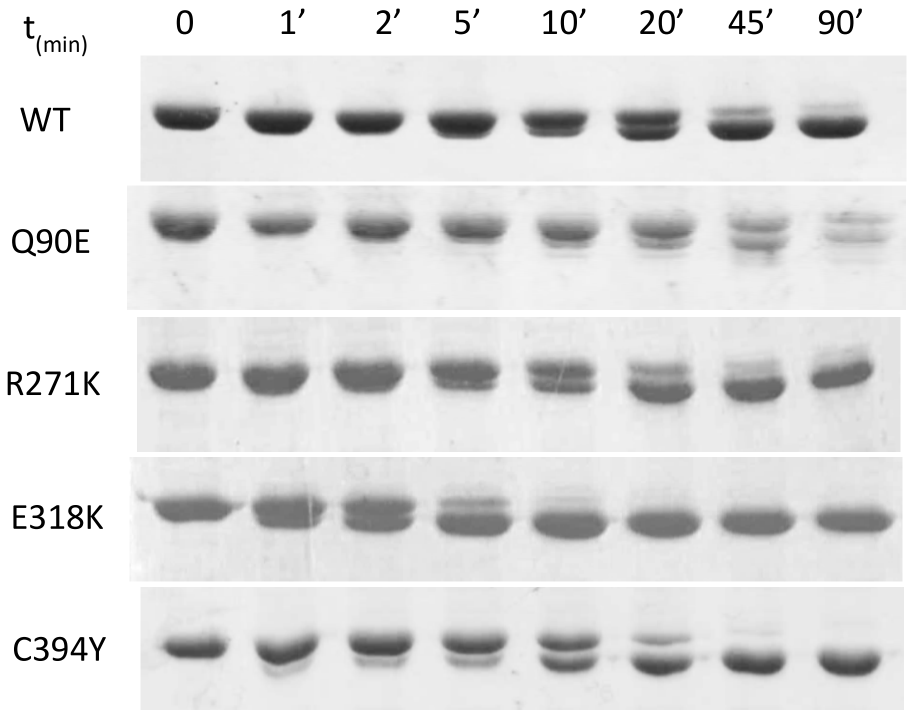

Fig. S7. Limited proteolysis with proteinase K. hOAT wt and the variants Q90E, R271K, E318K and C394Y were incubated with proteinase K at a 1:100 protease/mutant ratio (w/w) at 25°C in PBS buffer pH 8.0 in the presence of 20 µM PLP. At the indicated time aliquots were treated with PMSF and subjected to 12% SDS-PAGE analysis.

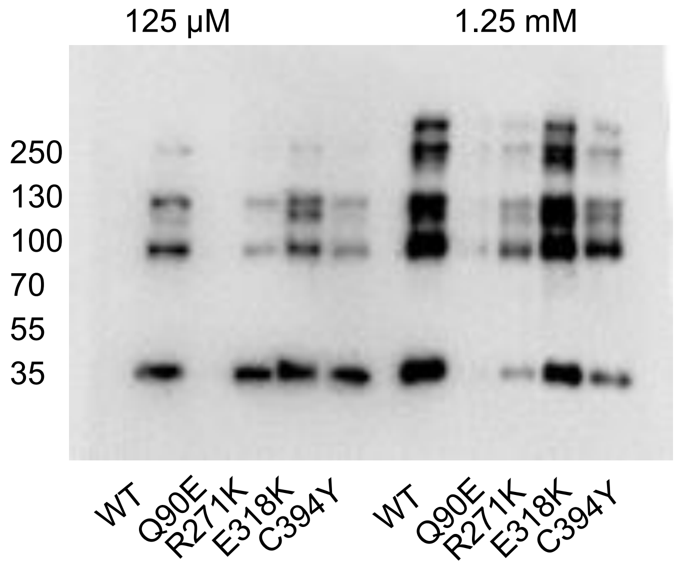

Fig. S8. Cross-linking analyses of hOAT wild-type and variants Hek293_OAT-KO cells expressing the indicated species were lysed and subjected to cross-linking with BS(PEG)5 at 125 µM or 1.25 mM concentration, as indicated, and then analyzed by western blot with an anti-hOAT antibody from mouse.

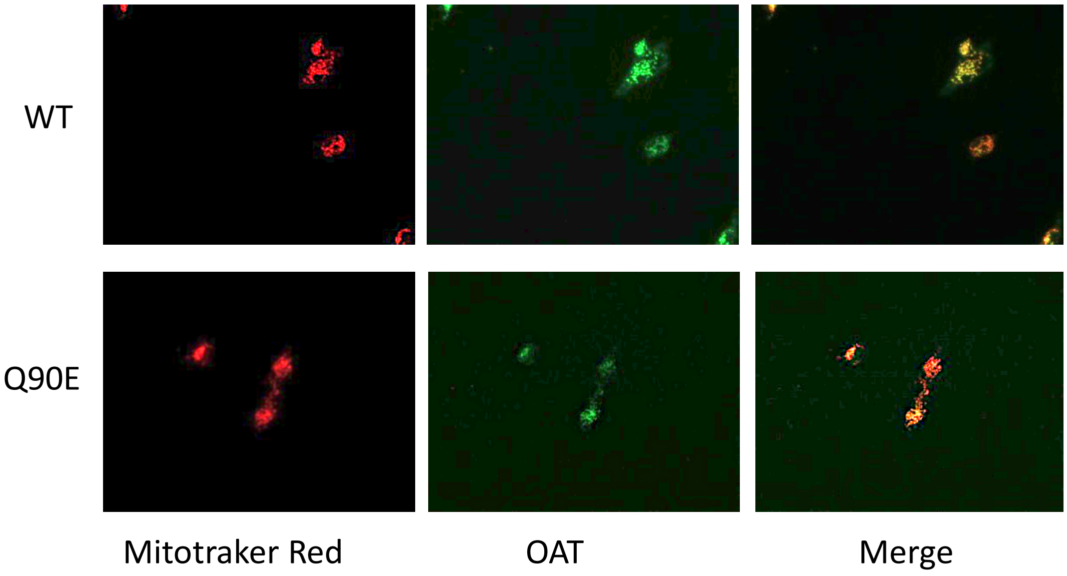

Fig. S9. Subcellular localization of hOAT wild-type and the Q90E variant. Hek293_OAT-KO cells expressing hOAT wild-type and the Q90E variant were fixed and stained with Mitotracked Red and an antibody against OAT (green) Nuclei were stained with Dapi (blue). Merge and single channel images come from a single z-plane.
